# Supplementary material for: Amorphous silicon-carbide photonics for ultrasound imaging
Source: Commun Phys. 2025 Dec 18;9(1):25. doi: 10.1038/s42005-025-02456-9 (PMC12819153; doi:10.1038/s42005-025-02456-9)
Supplement: Supplementary file 2 — Supplementary Information [file 42005_2025_2456_MOESM2_ESM.pdf]

Supplementary Information: Amorphous Silicon-Carbide Photonics for Ultrasound Imaging

R. Tufan Erdogan[1*], Bruno Lopez-Rodriguez[2], Wouter J. Westerveld[1], Sophinese Iskander-Rizk[1], Gerard J. Verbiest[1], Iman Esmaeil Zadeh[2], Peter G. Steeneken[1*]

[1]Department of Precision and Microsystems Engineering, TU Delft, The Netherlands.
[2]Department of Imaging Physics (ImPhys), TU Delft, The Netherlands.

*Corresponding author(s). E-mail(s): r.t.erdogan@tudelft.nl; p.g.steeneken@tudelft.nl;
Contributing authors: b.lopezrodriguez@tudelft.nl; w.j.westerveld@tudelft.nl; s.iskander-rizk@tudelft.nl; g.j.verbiest@tudelft.nl; i.esmaeilzadeh@tudelft.nl;

Supplementary Note 1: Sample holders for ultrasound imaging experiments

For imaging experiments we design and 3D print two type of holders. In Supplementary Figure 1a and Supplementary Figure 1b, photographs of printed parts are provided. The parts are designed such that the wires can be attached to the side of the holder and the holder can easily be attached to the aluminum chip holder as shown in Supplementary Figure 1c and Supplementary Figure 1d. The height of the the aluminum wire was approximately 500 µm, and two fibers were approximately at the height of 1 mm and 2 mm from the surface of the chip. However, due to the attractive forces when submerged under water the fiber that is placed at 2 mm distance from the chip deformed and got closer to the lower one. This was also evident from the photographs taken after the experiments that is shown in Supplementary Figure 1d.

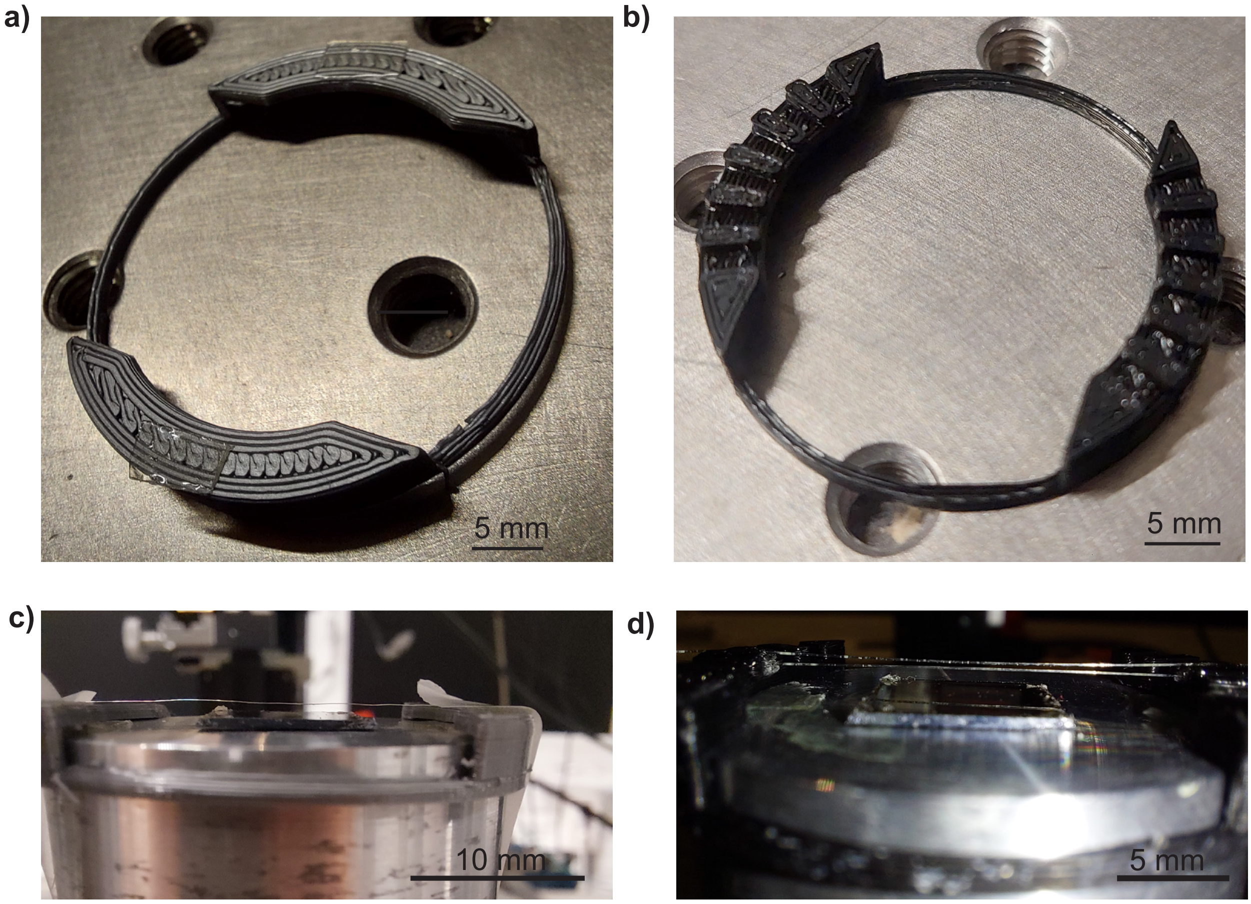

Supplementary Figure 1: Sample holders and fixture for the imaging experiments. **a)**, and **b)** aluminum wire holder and SM fiber holder, respectively. **c)**, and **d)** Placement of the holders shown in **(a)**, and **(b)** to the aluminum chip stage after the aluminum wire and SM fibers are attached and used in experiments.

Supplementary Note 2: Ultrasound sensitivity of the ring resonators

The voltage at the output, V_{out}, of the photoreceiver connected to the output of the ring resonator through-port can be written as:

$$V_{\text{out}}(\lambda_{\text{f}}, \partial\sigma) = \psi P_{\text{out}} T(\lambda_{\text{f}}, \partial\sigma) = \psi P_{\text{out}} T(\lambda_{\text{f}}) + \left.\frac{\partial V_{\text{out}}}{\partial \sigma}\right|_{\lambda_{\text{f}}} \partial\sigma \tag{1}$$

where $\psi = R_{PD}G_{TIA}$ is a constant composed of the photodiode responsivity and the gain of the trans-impedance amplifier and $P_{\text{out}} = \eta_{gra}P_{laser}$ is the output power of the light in which η_{gra} refers to total coupling efficiency of the two grating couplers. The output voltage depends on the output light power, P_{out}, and the transmission function of the ring resonator at the flank wavelength (λ_f), $\left.T(\lambda_f, \partial\sigma)\right|_{\lambda_{\text{f}}}$. This transmission function can be expanded to include a perturbation term of pressure, $\partial\sigma$, that accounts for changes in the ultrasound pressure.

To quantify the total sensitivity of the sensor, S_{tot}, we can express it in terms of two distinct contributions relevant to the sensors' design and waveguide properties, S_{ph} and S_{int}, and other constants regarding the experimental setup:

$$S_{\text{tot}} = \left.\frac{\partial V_{\text{out}}}{\partial \sigma}\right|_{\lambda_{\text{f}}} = P_{\text{out}}\psi S_{\text{ph}} S_{\text{int}} \tag{2}$$

where P_{out} and ψ are the experimental setup related constants, S_{ph} is the sensitivity of photonic transduction of the transmission amplitude monitoring method, and S_{int} is the intrinsic sensitivity of the resonator as a change in wavelength to the applied pressure. Accordingly, the total sensitivity can be rewritten in terms of the derivative of the transmission function at the flank wavelength and resonance wavelength shift due to pressure:

$$S_{\text{tot}} = P_{\text{out}}\psi \left.\frac{\partial T}{\partial \lambda_{\text{res}}}\right|_{\lambda_{\text{f}}} \frac{\partial \lambda_{\text{res}}}{\partial \sigma} \tag{3}$$

The third term on the right side of Eq. 3, represents the photonic contribution to sensitivity:

$$S_{\text{ph}} = \left.\frac{\partial T}{\partial \lambda_{\text{res}}}\right|_{\lambda_{\text{f}}} \sim \frac{1}{FWHM} \sim \frac{Q}{\lambda_{\text{res}}} \tag{4}$$

the reciprocal of full width at half maximum (FWHM) characterizes the slope of the resonance transmission spectrum for high-quality factor resonators operated at the flank wavelength, and Q is the resonator's quality factor. The propagation losses in the ring resonator and the coupling between the bus waveguide and the ring waveguide determine the Q-factor of the resonator.

The last term on the right side of the Eq. 3, captures the intrinsic sensitivity of the ring waveguide, which quantifies the shift in resonance wavelength due to applied pressure. Since the core materials we consider here have high Young's modulus and the applied pressure amplitudes of photoacoustic signals are small, the

deformation of the waveguide core and change in the ring circumference can be ignored. In this case, only photoelastic changes in the refractive index of materials are considered:

$$S_{\text{int}} = \frac{\partial \lambda_{\text{res}}}{\partial \sigma} = \frac{\partial \lambda_{\text{res}}}{\partial n_{\text{eff}}} \frac{\partial n_{\text{eff}}}{\partial \sigma} \tag{5}$$

Expanding the dependence of the effective index on different material components of the waveguide:

$$S_{\text{int}} = \frac{\lambda_{\text{res}}}{n_{\text{g}}} \left(\frac{\partial n_{\text{eff}}}{\partial n_{\text{core}}} \frac{\partial n_{\text{core}}}{\partial \sigma} + \frac{\partial n_{\text{eff}}}{\partial n_{\text{cladding}}^{\text{top}}} \frac{\partial n_{\text{cladding}}^{\text{top}}}{\partial \sigma} + \frac{\partial n_{\text{eff}}}{\partial n_{\text{cladding}}^{\text{bottom}}} \frac{\partial n_{\text{cladding}}^{\text{bottom}}}{\partial \sigma} \right) \tag{6}$$

For different waveguide materials, different terms dominate the intrinsic sensitivity. The interaction coefficients, $\frac{\partial n_{\text{eff}}}{\partial n_X}$, can be determined with FEM simulations once the refractive index of the waveguide materials and the core geometry is known, where n_X refers to different material domains of the waveguide.

We consider two different cases. Case 1: core materials with low photoelasticity, for example, Silicon (Si), Silicon Nitride (SiN), and Amorphous Silicon Carbide (a-SiC) waveguide cores with a highly photoelastic top cladding material such as PDMS, where the top cladding dominates S_{int}.

In this scenario, the effective index change is primarily influenced by the top cladding:

$$S_{\text{int}} = \frac{\lambda_{\text{res}}}{n_{\text{g}}} \left(\frac{\partial n_{\text{eff}}}{\partial n_{\text{cladding}}^{\text{top}}} \frac{\partial n_{\text{cladding}}^{\text{top}}}{\partial \sigma} \right) \tag{7}$$

Thus, the total sensitivity simplifies to:

$$S_{\text{tot}} = \psi P_{\text{out}} \frac{Q}{n_{\text{g}}} \left(\frac{\partial n_{\text{eff}}}{\partial n_{\text{cladding}}^{\text{top}}} \frac{\partial n_{\text{cladding}}^{\text{top}}}{\partial \sigma} \right) \tag{8}$$

Case 2: core materials with high photoelasticity, such as chalcogenide waveguide core, where core photoelasticity is comparable to that of PDMS top cladding photoelasticity.

In this case, since the waveguide mode is confined in the core material, only the core photoelasticity dominates the S_{int} and cladding photoelasticity can be ignored:

$$S_{\text{int}} = \frac{\lambda_{\text{res}}}{n_{\text{g}}} \left(\frac{\partial n_{\text{eff}}}{\partial n_{\text{core}}} \frac{\partial n_{\text{core}}}{\partial \sigma} \right) \tag{9}$$

leading to the total sensitivity:

$$S_{\text{tot}} = \psi P_{\text{out}} \frac{Q}{n_{\text{g}}} \left(\frac{\partial n_{\text{eff}}}{\partial n_{\text{core}}} \frac{\partial n_{\text{core}}}{\partial \sigma} \right) \tag{10}$$

Eq.(10) and Eq.(8) show that ψ, P_{out}, Q, and n_{g} are the scaling factors that affect the sensitivity for all kinds of waveguides in ultrasound sensing, and the core and cladding material photoelasticity determines the dominating factor of S_{int} in ultrasound sensing.

We used a semi-analytical waveguide model [1] to calculate n_{eff} of different waveguides, approximated Eq. 7 and Eq. 9 for different platforms and considered PDMS top cladding for each waveguide cross-section. The results of these calculations are given in Supplementary Table 1. We observe that if the sensing is dominated by the photoelasticity of the cladding material, S_{int} increases as the optical confinement of the mode decreases. Since the minimum bending radius of the waveguide increases as the optical confinement decreases, the result suggests that there is a trade-off between S_{int} and the miniaturization potential of ring resonators. On the other hand, if the core material's photoelasticity dominates the sensitivity, then the overall sensitivity is higher than the waveguides, where the cladding photoelasticity is the dominating factor.

	Waveguide core material				
	Si	a-SiC	SiN	ChG	Unit
Core dimensions	450 × 220	800 × 280	1000 × 400	2400 × 850	nm×nm
n_{core}	3.476 [2]	2.55 [3]	1.996 [2]	2.33 [4]	RIU
n_{eff}	2.3407	1.9030	1.608	2.203	RIU
n_{g}	4.262	2.789	2.096	2.408	RIU
$\frac{\partial n_{\text{eff}}}{\partial \lambda} \times 10^3$	-1.24	-0.572	-0.315	-0.132	RIU/nm
$\frac{\partial n_{\text{eff}}}{\partial n_{\text{core}}}$	1.136	0.961	0.827	1.018	RIU/RIU
$\frac{\partial n_{\text{eff}}}{\partial n_{\text{cladding}}^{\text{top}}}$	0.17	0.211	0.272	0.0234	RIU/RIU
$\frac{\partial n_{\text{core}}}{\partial P}$	n.a.	n.a.	n.a.	545* [5]	RIU/TPa
$\frac{\partial n_{\text{cladding}}^{\text{top}}}{\partial P}$	664 [6]	664 [6]	664 [6]	664 [6]	RIU/TPa
$\frac{\lambda_{\text{res}}}{n_{\text{g}}}$	364	556	740	644	nm/RIU
S_{int}	40	78	134	410	fm/kPa

Supplementary Table 1: Theoretical calculation of intrinsic sensitivity for different waveguide cores with a highly elasto-optic polymer cladding such as PDMS. All calculations are made based on the TE0-like waveguide mode and at 1550 nm.
*Calculated using the values given for C_R and C_θ in [5]
n.a. : not applicable since Eq. 7 is used for the S_{int} calculation.
RIU : refractive index unit

Supplementary Note 3: k-Wave Simulations of Ultrasound Imaging Experiments

In this section, using the k-wave [7] add-on in MATLAB, we explain how the imaging in our setup is performed. The simulation and experiment results agree well regarding the time of flight between the initial pulse arrival and the signal from the samples.

In these simulations, $dx = dy = 3.85\,\mu\text{m}$ and $dt = 0.125\,\text{ns}$ is defined as the k-wave grid parameters. Speed of sound is used as $1540\,\text{m}\,\text{s}^{-1}$. A longitudinal wave equivalent pulse is defined at an angle representing the experiments. The speed of sound and the density of the single aluminum wire or the double fibers are defined at the approximate positions, which are found as a result of the imaging experiments (main text Fig. 5g, 5h). For both of the simulations, the grid parameters were kept the same; the simulation time window is adjusted according to the experiments.

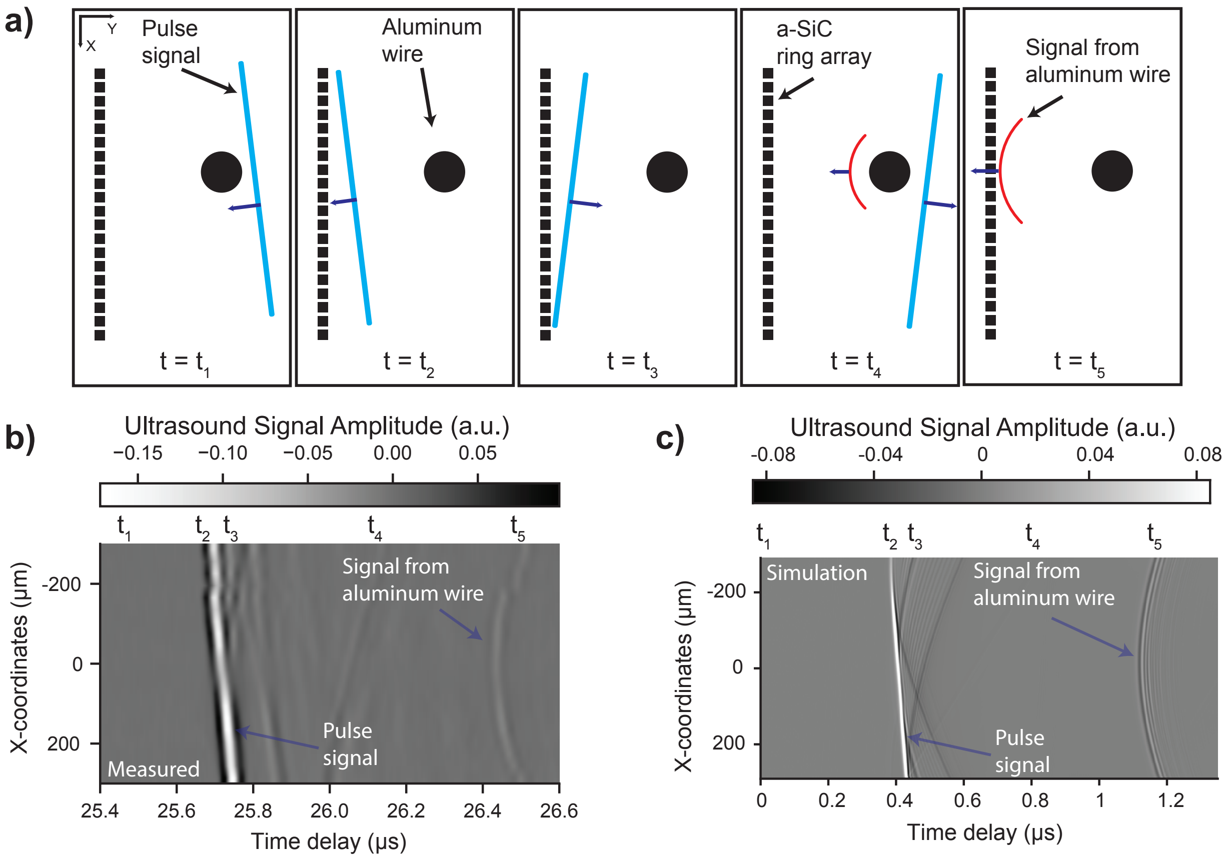

Supplementary Figure 2: Comparison of the imaging experiments and k-wave simulations for the aluminum wire imaging experiment. **a)** schematic demonstration of the wave propagation in the water tank for different timestamps. **b)** Measured data using the linear array of 20 a-SiC ring resonators. **c)** Recorded amplitude data in the simulation by the point detectors.

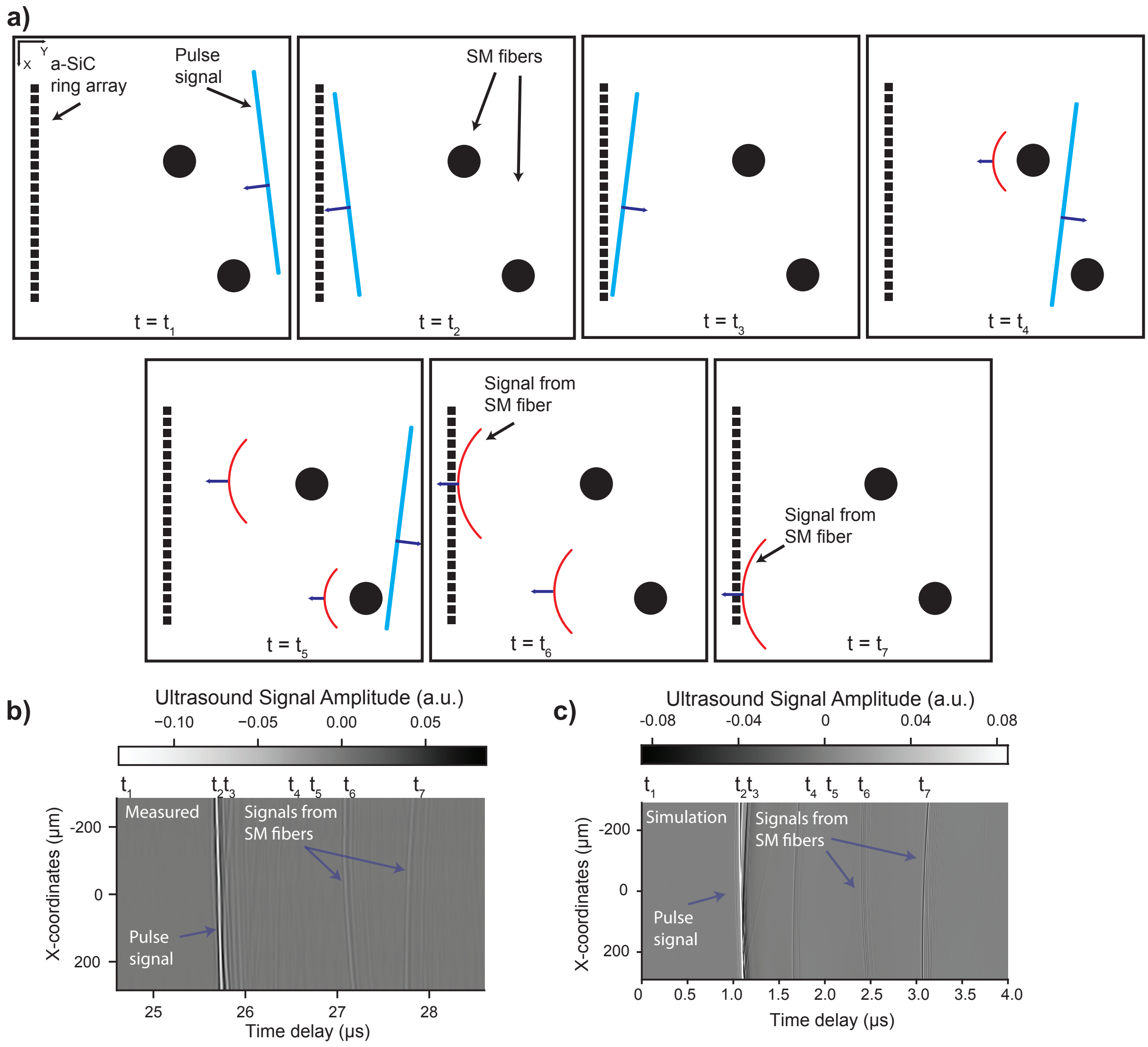

Supplementary Figure 3: Comparison of the imaging experiments and k-wave simulations for the two SM fiber imaging experiments. **a)** schematic demonstration of the wave propagation in the water tank for different timestamps. **b)** Measured data using the linear array of 20 a-SiC ring resonators. **c)** Recorded amplitude data in the simulation by the point detectors.

Supplementary Note 4: Pressure calibration using a hydrophone

In this section, we expand on the details of the calibration of the pressures in our setup and the calculation method of the NEPD of the sensors. A calibrated needle hydrophone (0.075 mm, Needle Hydrophone, Precision Acoustics, SN:1302) is used to calibrate the pressure generated in our setup through the use of the immersion ultrasound transducer (V316-SM, Evident). The sensor and the hydrophone were placed at the same depth in both the calibration and NEPD characterization experiments. The in-plane position of the hydrophone of the sensor is optimized for the maximum amplitude received by the sensor or the hydrophone. A Gaussian signal is generated through the use of an AWG (Supplementary Figure 4a), and the same pulse is used in both experiments. The recorded signal using the hydrophone is plotted in Supplementary Figure 4b, and the FFT of this signal is plotted in Supplementary Figure 4c and Supplementary Figure 4d. FFT amplitude is converted to dB scale in Supplementary Figure 4d to visualize the calibration frequency high and low cut-off values. The calibration bandwidth is limited to the frequency cut-off values where the pressure signal is found to be 10 dB less than the highest pressure signal generated by the ultrasound transducer for the lower cut-off, and the highest frequency of the calibration data of the hydrophone is used as the upper cut-off. Sensitivity data (Supplementary Figure 4e) of the hydrophone from the calibration certificate is used to calculate the pressure spectrum at the location of the hydrophone. Since the calibration data is sampled at 1 MHz, we fit a polynomial to this data to smooth the results. The calculated pressure spectrum is plotted in Supplementary Figure 4f and further used in the a-SiC ultrasound sensors' NEPD calculations.

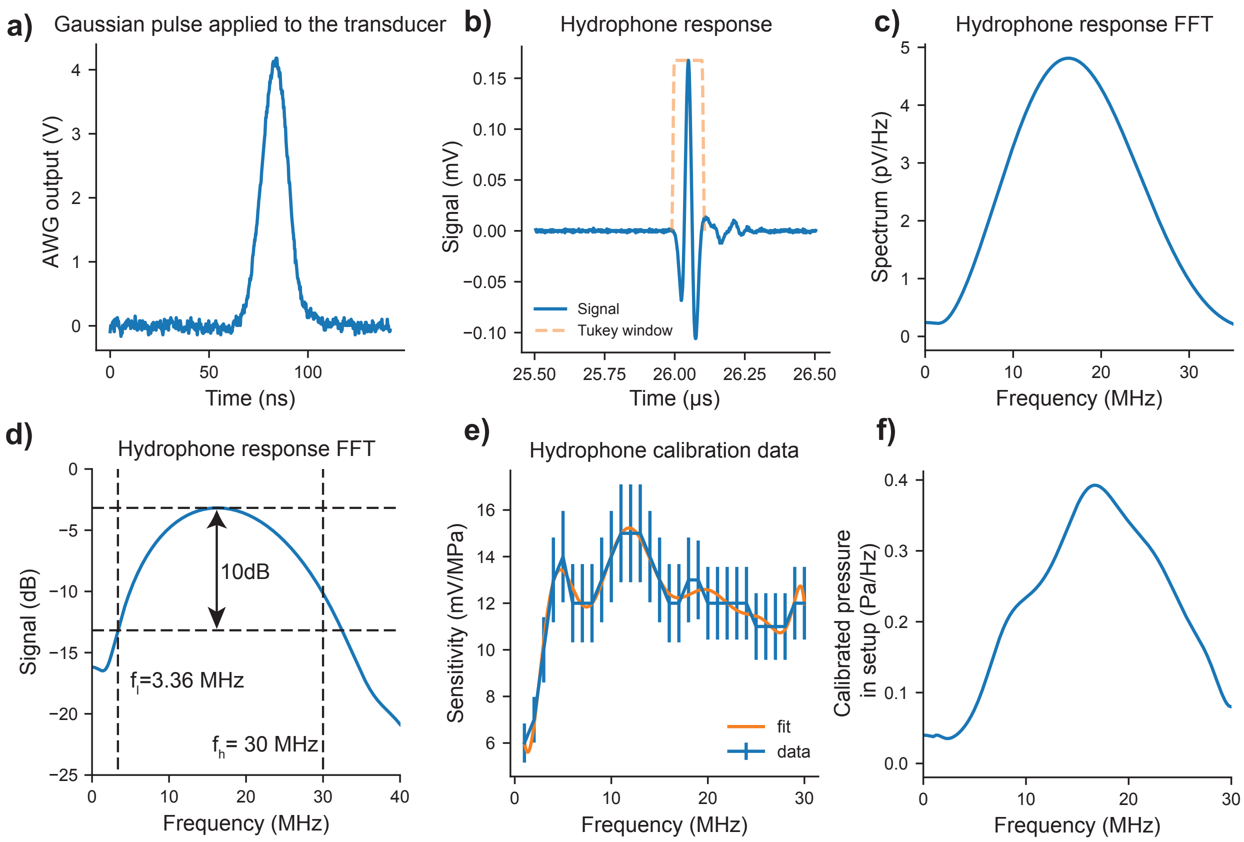

Supplementary Figure 4: Pressure calibration of the ultrasound transducer. **a)** The Gaussian pulse applied to the ultrasound transducer for pressure calibration of the transducer. Measured from the output of the arbitrary waveform generator (AWG). **b)** The measured signal from the hydrophone, which is placed across the ultrasound transducer at a distance greater than the near field of the ultrasound transducer. **c)** FFT of the hydrophone signal shown in **(b)**. **d)** The calibration bandwidth determination from the FFT result of the hydrophone. **e)** The calibration data of the hydrophone, supplied by Precision Acoustics ltd. A fit is performed to interpolate the data points for further calculations of the NEPD and pressures. **f)** The pressure spectrum in the setup calculated using **(c)** and **(e)**.

Supplementary Note 5: NEPD calculation of the a-SiC ultrasound sensor

The NEPD calculation of the sensors in our study follows the earlier work in the field [4, 8]. To characterize the a-SiC sensors NEPD in the bandwidth of the calibration we used the same Gaussian signal described in Supplementary Figure 4a, placed the sensor at the same depth as the hydrophone, and recorded the ultrasound response of the sensor. The response to the Gaussian pulse is plotted in Supplementary Figure 2a and its FFT is given in Supplementary Figure 4b. Using the frequency response of the sensor and the calibrated pressure spectrum from Supplementary Figure 4f responsivity of the a-SiC ring resonator is calculated and the result is plotted in Supplementary Figure 2c. Following the recording of the ultrasound signal with the sensor, the noise is recorded at the same wavelength and laser power as the ultrasound recordings. No averaging and no ultrasound signal is applied while recording the noise signal. This noise recording is plotted Supplementary

Figure 2d and its noise amplitude spectral density (ASD) (Supplementary Figure 2e) of estimated using the Welch method with segments of 2048 samples, overlapping of 512 samples and using a Hanning window. By dividing the ASD (Supplementary Figure 2e) to the responsivity (Supplementary Figure 2c) of the sensor, the NEPD is calculated and plotted (Supplementary Figure 2f). The maximum NEPD of the sensor in the range of calibration is found to be 55 mPa/$\sqrt{\text{Hz}}$ and the mean value is found to be 45 mPa/$\sqrt{\text{Hz}}$. The characteristic distribution of the hydrophone calibration data given in Supplementary Figure 4e resembles itself in the NEPD distribution of the senor. This observation suggests that the sensor's response is approximately flat over the range of calibration frequencies.

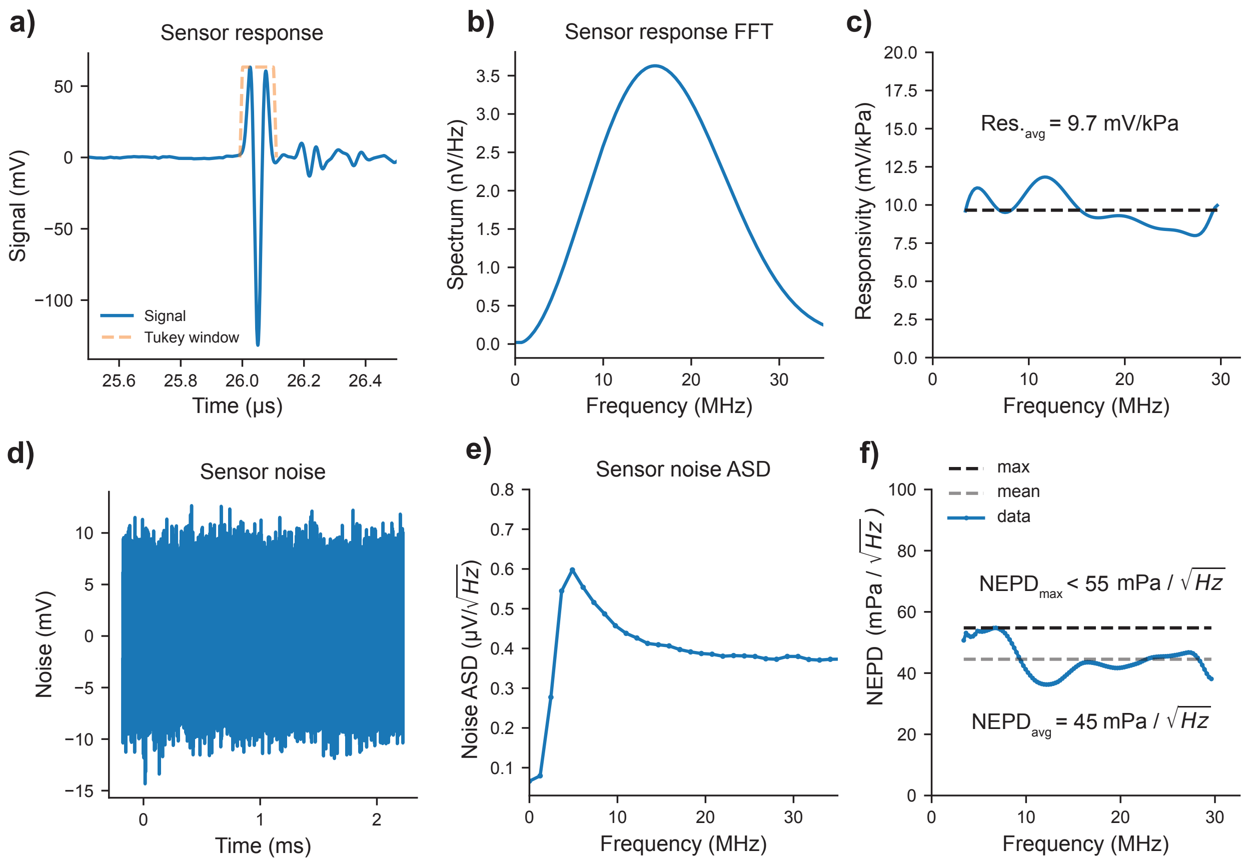

Supplementary Figure 5: NEPD calculation of the a-SiC ring resonator. **a)** The measured signal from the a-SiC microring resonator represented in the main text Fig. 3, which is placed across the ultrasound transducer at the same distance as the hydrophone. **b)** FFT of the signal shown in **(a)**. **c)** The calibrated responsivity function of the a-SiC microring resonator in ultrasound sensing. **d)** Recorded noise signal without averaging or any ultrasound pressure application. **e)** The noise amplitude spectral density (ASD) of the signal shown in **(d)**. The Welch method is used to calculate the ASD with a Hanning window with segments of 2,048 samples, 512 samples overlapping, and a linear detrend. **f)** The noise equivalent pressure density of the a-SiC microring resonator is calculated using the responsivity spectrum and noise amplitude spectral density calculation. The mean and maximum value of the NEPD are also annotated with dashed lines and text.

Supplementary Note 6: Determination of the lateral position of the ring resonators on the chip

In the main text, we annotate the ultrasound measurement range of wavelengths with the array of ring resonators in Fig. 4b. The ultrasound data with each ring resonator in the array is collected from the indicated 20 notches, sequentially. However, the order of the resonance frequencies differs from the placement order of the ring resonators in the chip. This can happen due to the variations in the fabrication during the EBL exposure process, unequal etching, or non-uniformity in the cladding PDMS layer. We correct the order of recorded ultrasound signals and determine the position of the ring resonators by using the two ultrasound experiments as depicted in Supplementary Figure 2a, a recording of the ultrasound pulse without a sample in the water, and Supplementary Figure 2b, the recording of the ultrasound pulse with the aluminum wire between the ultrasound transducer and the ring array. Supplementary Figure 2c and Supplementary Figure 2d are the recorded ultrasound signals from the ring resonator array, where the resonator index is given as the measurement order (i.e., resonance wavelength order). Both of the recordings show that, according to the initial pulse arrival time, the ring resonators from index 16 to 19 receive the pulse earlier than expected, suggesting that their placement order in the chip does not follow their resonance wavelength; hence, their index needs to be corrected. Accordingly, we sort the indices of recorded responses given in Supplementary Figure 2c and Supplementary Figure 2d based on the initial pulse arrival time in Supplementary Figure 2e and Supplementary Figure 2f, respectively. Furthermore, we realize that the initial pulse arrival sorting is not enough to correctly sort the indices of the two resonators, namely the resonator at indices 4 and 5, in Supplementary Figure 2e and Supplementary Figure 2f. As indicated in the figures with the red arrow, the time of receiving the reflection signals of the initial ultrasound pulses for these resonators creates a discontinuity in the signal recorded, which suggests a surface defect near the location of the resonators that is causing the signal to be delayed further than uniform over the whole array. According to this observation, we switch the indices of these two resonators and reach the final, correctly sorted indices of the whole array. Supplementary Figure 2g and Supplementary Figure 2h plot the resulting final data from both of the experiments depicted in Supplementary Figure 2a and Supplementary Figure 2b, respectively.

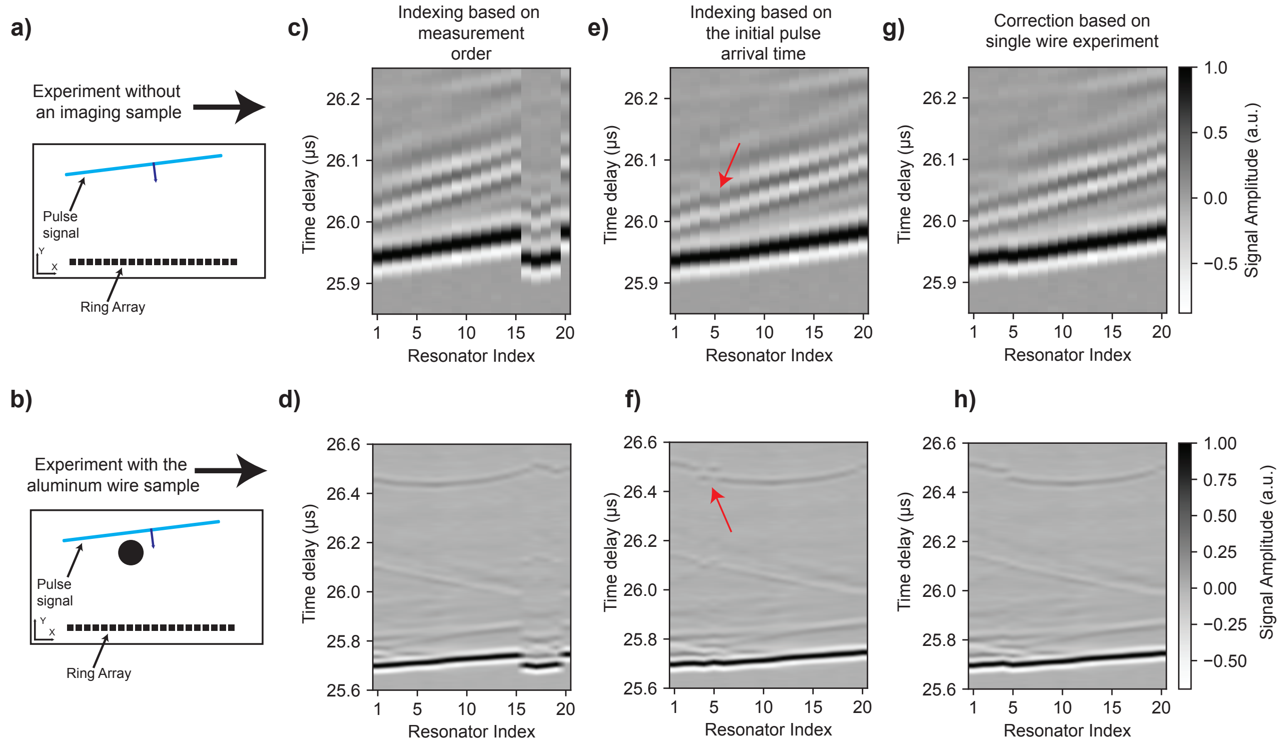

Supplementary Figure 6: Sorting of the array of ring resonators using the experimental data with and without an aluminum wire to determine the location of the ring resonators on the chip. **a**) Schematic cross-sectional view of the pulse sensing experiment with the ring resonator array. **b**) Schematic cross-sectional view of the imaging of the aluminum wire experiment with the ring resonator array. **c**) and **d**) The recorded ultrasound signals, which are sorted based on the notch wavelength order in the time domain for the experiments shown in (**a**) and (**b**), respectively. **e**) and **f**) The time traces in (**c**) and (**d**) are sorted based on the initial pulse arrival time. The discrepancy in the reflection signals is indicated with red arrows. **g**) and **h**) The time traces after the final adjustment based on the reflection signals in (**e**) and (**f**)

Supplementary References

[1] Westerveld, W. J., Leinders, S. M., van Dongen, K. W. A., Urbach, H. P. & Yousefi, M. Extension of marcatili's analytical approach for rectangular silicon optical waveguides. *Journal of Lightwave Technology* **30**, 2388–2401 (2012).

[2] Polyanskiy, M. N. Refractiveindex.info database of optical constants. *Scientific Data* **11**, 94 (2024). URL https://www.nature.com/articles/s41597-023-02898-2.

[3] Lopez-Rodriguez, B. *et al.* High-quality amorphous silicon carbide for hybrid photonic integration deposited at a low temperature. *ACS Photonics* **10**, 3748–3754 (2023). URL https://doi.org/10.1021/acsphotonics.3c00968.

[4] Pan, J. *et al.* Parallel interrogation of the chalcogenide-based micro-ring sensor array for photoacoustic tomography. *Nature Communications* **14**, 3250 (2023). URL https://www.nature.com/articles/s41467-023-39075-3.

[5] Bao, K. *et al.* Photoacoustic imaging sensors based on integrated photonics: Challenges and trends. *Laser & Photonics Reviews* **19**, 2400414 (2024). URL https://onlinelibrary.wiley.com/doi/abs/10.1002/lpor.202400414.

[6] Park, J. S., Cabosky, R., Ye, Z. & Kim, I. I. Investigating the mechanical and optical properties of thin pdms film by flat-punched indentation. *Optical Materials* **85**, 153–161 (2018). URL https://www.sciencedirect.com/science/article/pii/S0925346718305895.

[7] Treeby, B. E. & Cox, B. T. k-Wave: MATLAB toolbox for the simulation and reconstruction of photoacoustic wave fields. *Journal of Biomedical Optics* **15**, 021314 (2010). URL https://doi.org/10.1117/1.3360308.

[8] Westerveld, W. J. *et al.* Sensitive, small, broadband and scalable optomechanical ultrasound sensor in silicon photonics. *Nature Photonics* **15**, 341–345 (2021). URL https://www.nature.com/articles/s41566-021-00776-0.
