# Supplementary material for: Amorphous silicon-carbide photonics for ultrasound imaging
Source: Commun Phys. 2025 Dec 18;9(1):25. doi: 10.1038/s42005-025-02456-9 (PMC12819153; doi:10.1038/s42005-025-02456-9)
Supplement: Supplementary file 3 — Description of additional supplementary file [file 42005_2025_2456_MOESM3_ESM.pdf]

Description of additional supplementary file

File name: Supplementary Data

Description: contains the data for each figure in a separate tab
